# Supplementary figures and images for: Long-term anti-SARS-CoV-2 antibody trajectories after neutralizing monoclonal antibody treatment
Source: PLoS One. 2025 Jun 18;20(6):e0325561. doi: 10.1371/journal.pone.0325561 (PMC12176123; doi:10.1371/journal.pone.0325561)

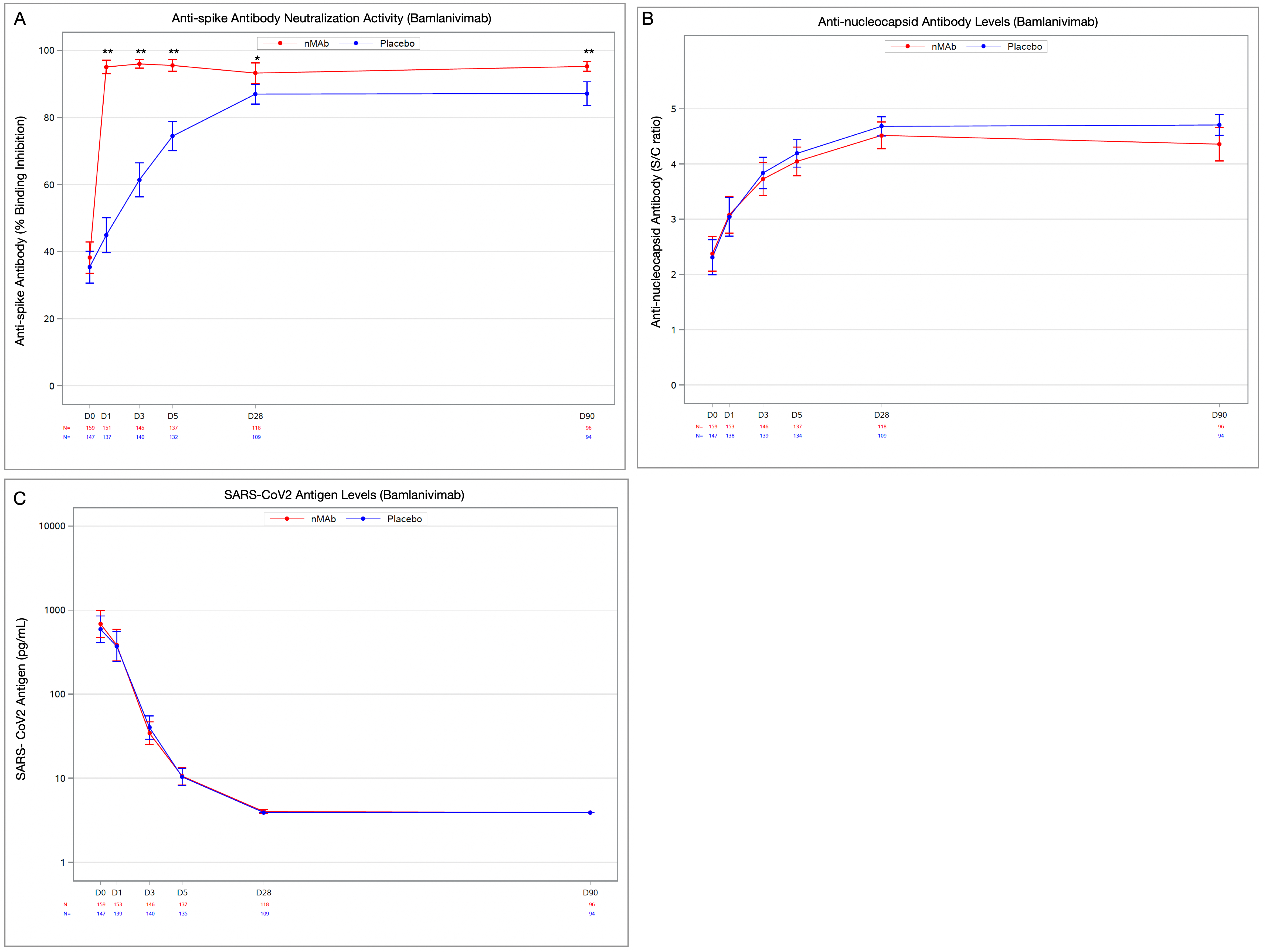

Supplement: S1 Fig — Antibody and antigen responses for the randomized controlled trial comparing treatment with the neutralizing monoclonal antibody Bamlanivimab (LILLY) vs placebo. Panel A: Anti-SARS-CoV-2 spike protein neutralization activity presented as percent binding inhibition (GenScript, Piscataway, New Jersey), Panel B: Total immunoglobulin (all immunoglobulin types) against the SARS-CoV-2 nucleocapsid antigen presented as signal-to-cutoff ratio (BioRad, Hercules, California), Panel C: SARS-CoV-2 nucleocapsid antigen levels presented as pg/mL on a log scale (Quanterix, Billerica, MA). * = p-value < 0.05; ** = p-value <0.001. (TIFF) [file pone.0325561.s001.tiff]

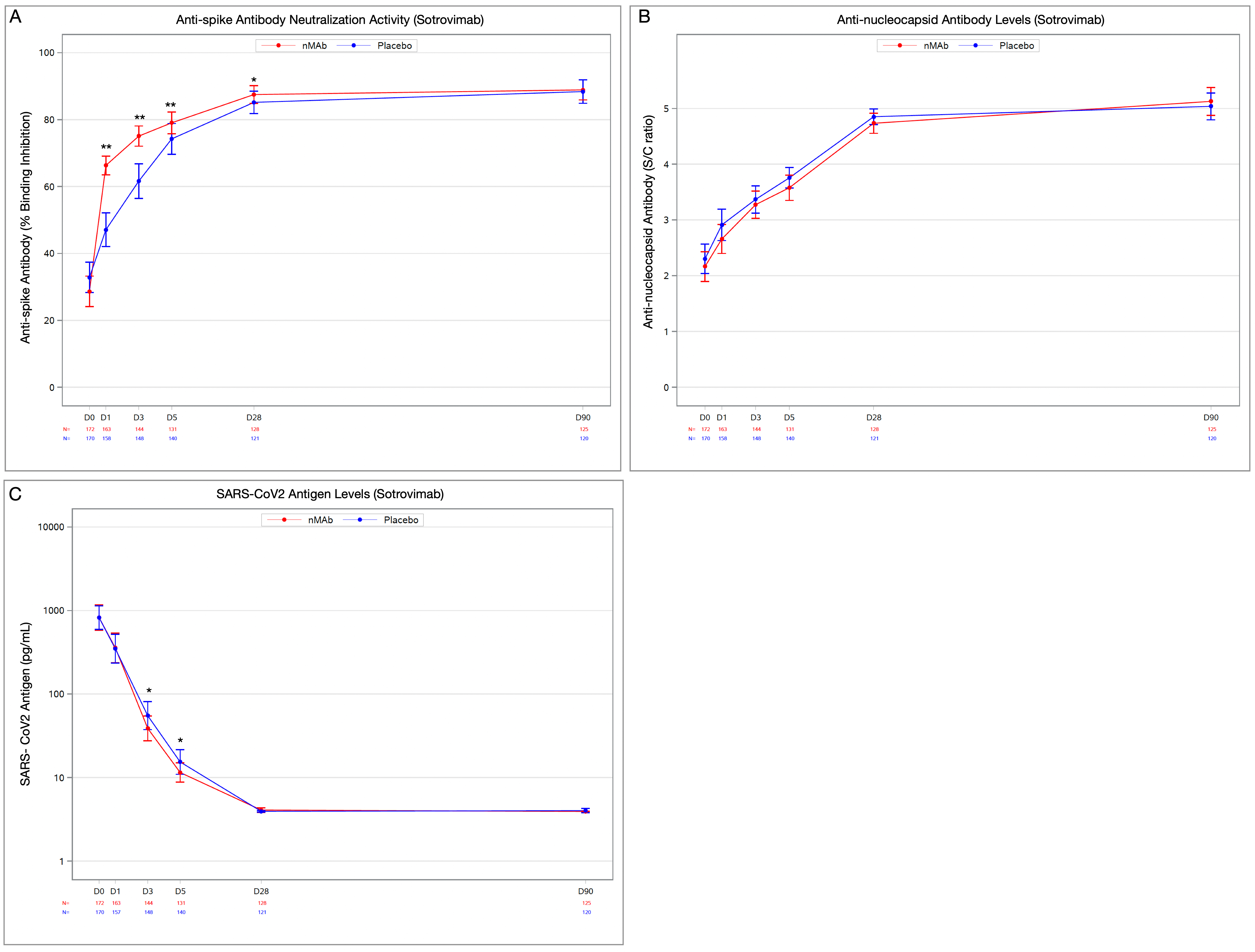

Supplement: S2 Fig — Antibody and antigen responses for the randomized controlled trial comparing treatment with the neutralizing monoclonal antibody Sotrovimab (VIR) vs placebo. Panel A: Anti-SARS-CoV-2 spike protein neutralization activity presented as percent binding inhibition (GenScript, Piscataway, New Jersey). Of note, Sotrovimab, which blocks viral fusion, recognizes a proteoglycan epitope distinct from the receptor binding motif itself and therefore is not detected by this GenScript assay. Panel B: Total immunoglobulin (all immunoglobulin types) against the SARS-CoV-2 nucleocapsid antigen presented as signal-to-cutoff ratio (BioRad, Hercules, California). Panel C: SARS-CoV-2 nucleocapsid antigen levels presented as pg/mL on a log scale (Quanterix, Billerica, MA). * = p-value < 0.05; ** = p-value <0.001. (TIFF) [file pone.0325561.s002.tiff]

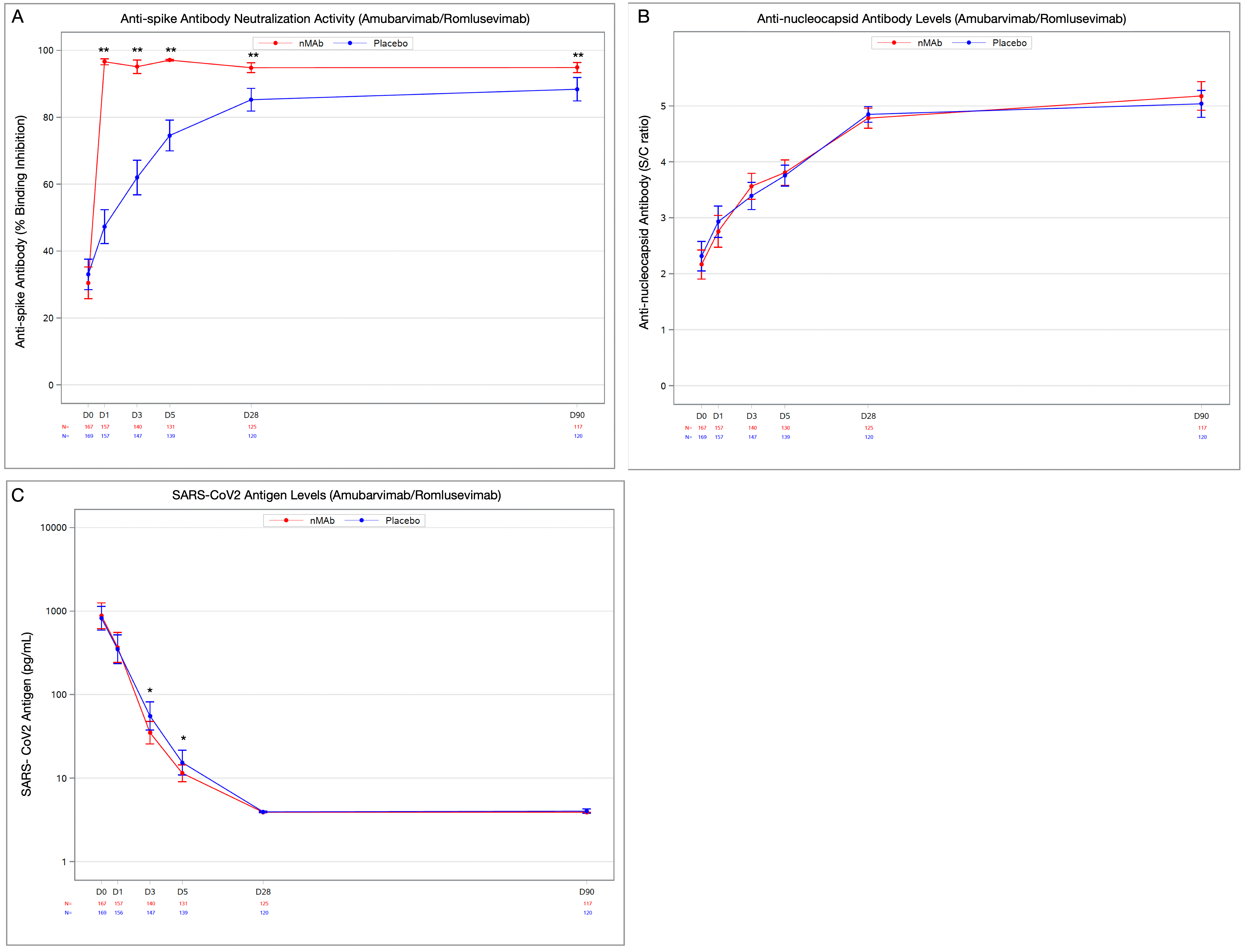

Supplement: S3 Fig — Antibody and antigen responses for the randomized controlled trial comparing treatment with the neutralizing monoclonal antibody Amubarvimab/ Romlusevimab (BRII) vs placebo. Panel A: Anti-SARS-CoV-2 spike protein neutralization activity presented as percent binding inhibition (GenScript, Piscataway, New Jersey), Panel B: Total immunoglobulin (all immunoglobulin types) against the SARS-CoV-2 nucleocapsid antigen presented as signal-to-cutoff ratio (BioRad, Hercules, California), Panel C: SARS-CoV-2 nucleocapsid antigen levels presented as pg/mL on a log scale (Quanterix, Billerica, MA). * = p-value < 0.05; ** = p-value <0.00. (TIFF) [file pone.0325561.s003.tiff]

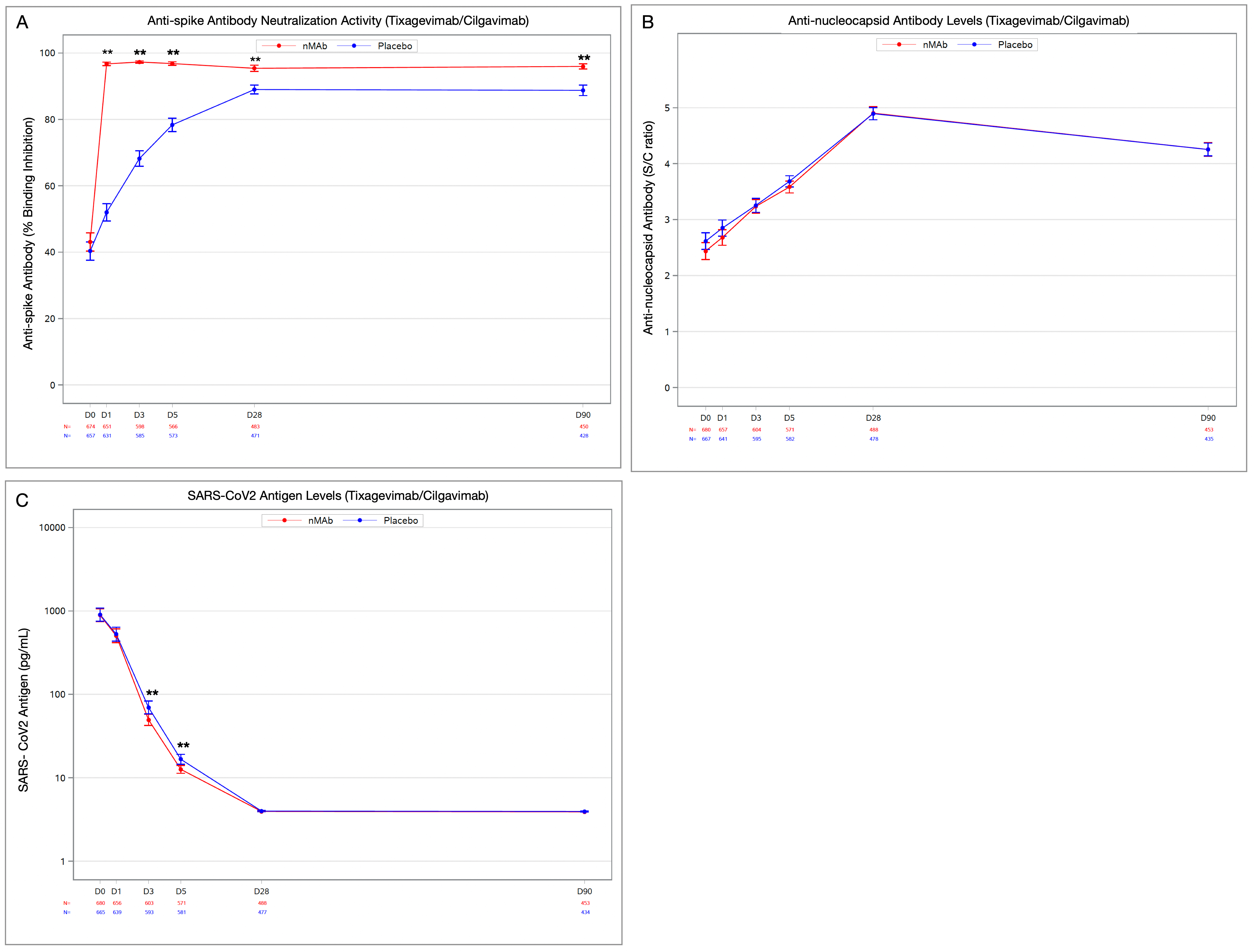

Supplement: S4 Fig — Antibody and antigen responses for the randomized controlled trial comparing treatment with the neutralizing monoclonal antibody Tixagevimab/Cligavimab (AZ) vs placebo. Panel A: Anti-SARS-CoV-2 spike protein neutralization activity presented as percent binding inhibition (GenScript, Piscataway, New Jersey), Panel B: Total immunoglobulin (all immunoglobulin types) against the SARS-CoV-2 nucleocapsid antigen presented as signal-to-cutoff ratio (BioRad, Hercules, California), Panel C: SARS-CoV-2 nucleocapsid antigen levels presented as pg/mL on a log scale (Quanterix, Billerica, MA). * = p-value < 0.05; ** = p-value <0.001. (TIFF) [file pone.0325561.s004.tiff]

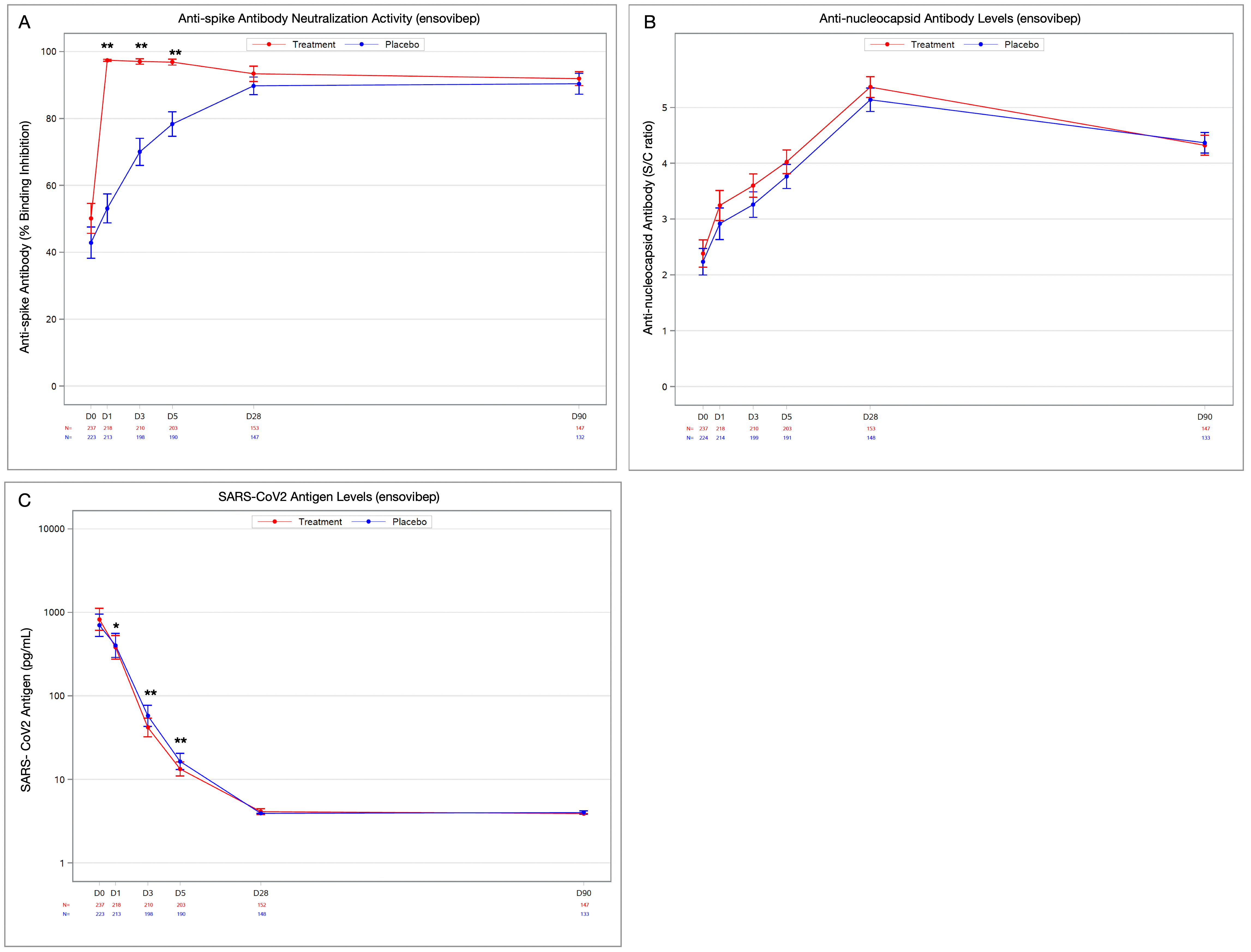

Supplement: S5 Fig — Antibody and antigen responses for the randomized controlled trial comparing treatment with ensovibep (MP) vs placebo. Ensovibep (MP) is a designed ankyrin repeat protein (DARPin) which targets and neutralizes the SARS-CoV-2 spike protein. Panel A: Anti-SARS-CoV-2 spike protein neutralization activity presented as percent binding inhibition (GenScript, Piscataway, New Jersey), Panel B: Total immunoglobulin (all immunoglobulin types) against the SARS-CoV-2 nucleocapsid antigen presented as signal-to-cutoff ratio (BioRad, Hercules, California), Panel C: SARS-CoV-2 nucleocapsid antigen levels presented as pg/mL on a log scale (Quanterix, Billerica, MA). * = p-value < 0.05; ** = p-value <0.001. (TIFF) [file pone.0325561.s005.tiff]

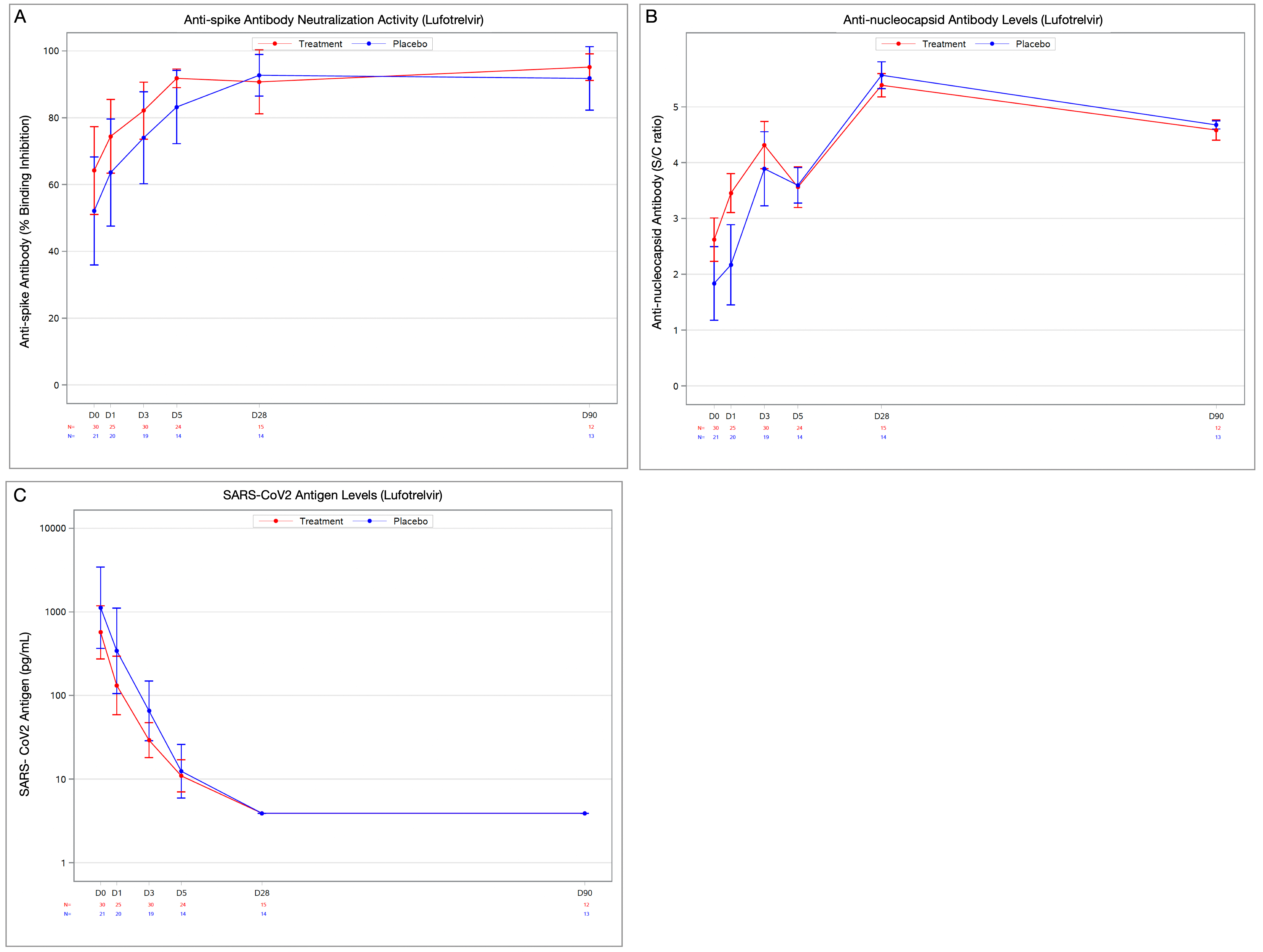

Supplement: S6 Fig — Antibody and antigen responses for the randomized controlled trial comparing treatment with lufotrelvir (PF) vs placebo. Lufotrelvir (PF) is a phosphate ester pro-drug that is a selective inhibitor of the SARS-CoV-2 3CLpro, a viral proteinase. Panel A: Anti-SARS-CoV-2 spike protein neutralization activity presented as percent binding inhibition (GenScript, Piscataway, New Jersey). Of note, Lufotrelvir targets viral assembly and therefore is not detected by this assay. Panel B: Total immunoglobulin (all immunoglobulin types) against the SARS-CoV-2 nucleocapsid antigen presented as signal-to-cutoff ratio (BioRad, Hercules, California). Panel C: SARS-CoV-2 nucleocapsid antigen levels presented as pg/mL (Quanterix, Billerica, MA). P-values not presented given small sample size (N = 51 at day 0, N = 25 at day 90). (TIFF) [file pone.0325561.s006.tiff]

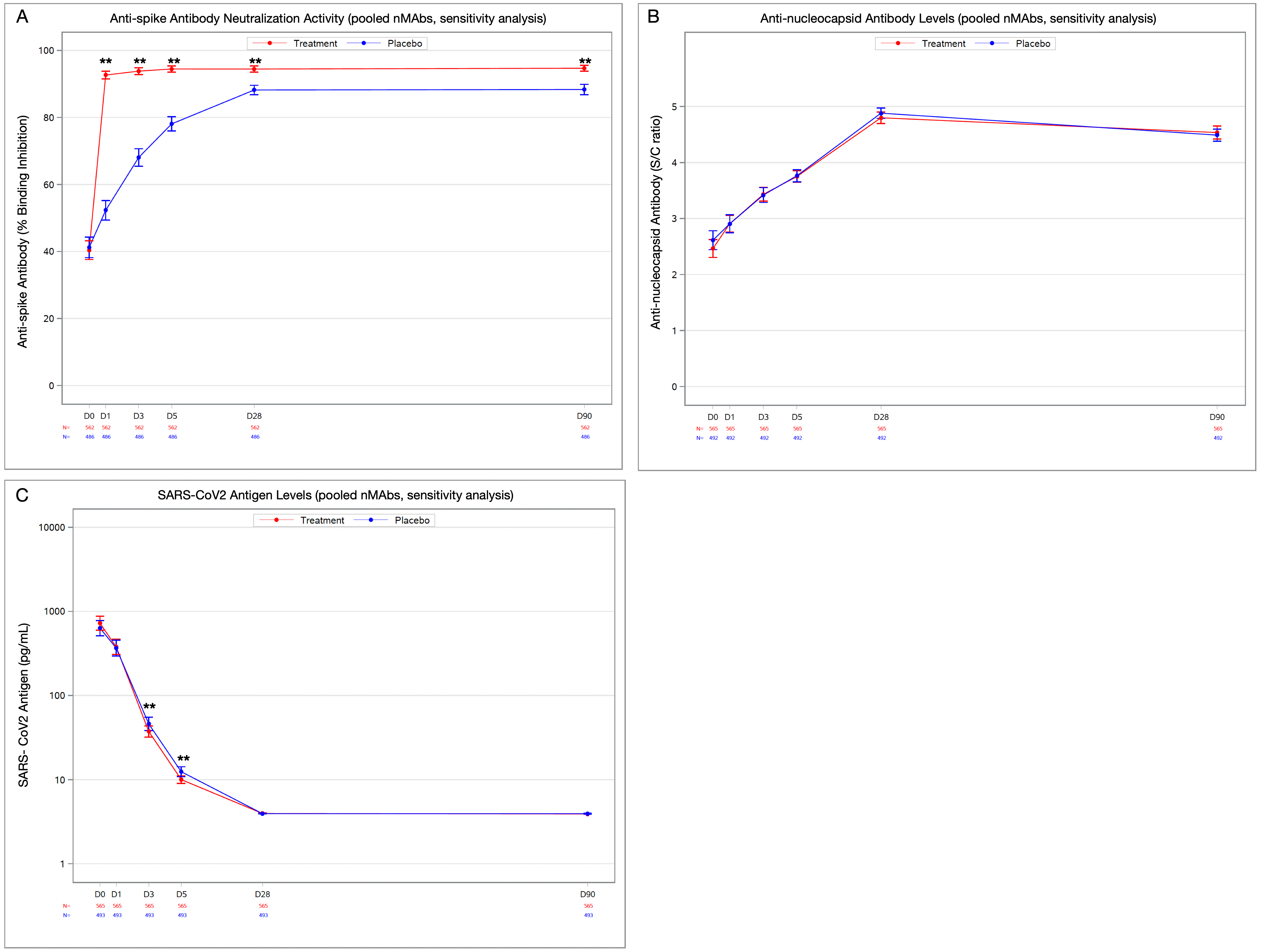

Supplement: S7 Fig — Pooled antibody and antigen responses for randomized controlled trials comparing treatment with neutralizing monoclonal antibodies (nMAbs) to placebo among patients alive and with all antibody/antigen measurements available at day 90. NMAbs included: Bamlanivimab (LILY), Sotrovimab (VIR), Amubarvimab/ Romlusevimab (BRII), Tixagevimab/ Cilgavimab (AZ). Panel A: Anti-SARS-CoV-2 spike protein neutralization activity presented as percent binding inhibition (GenScript, Piscataway, New Jersey), Panel B: Total immunoglobulin (all immunoglobulin types) against the SARS-CoV-2 nucleocapsid antigen presented as signal-to-cutoff ratio (BioRad, Hercules, California), Panel C: SARS-CoV-2 nucleocapsid antigen levels presented as pg/mL on a log scale (Quanterix, Billerica, MA). * = p-value < 0.05; ** = p-value <0.001. (TIFF) [file pone.0325561.s007.tiff]

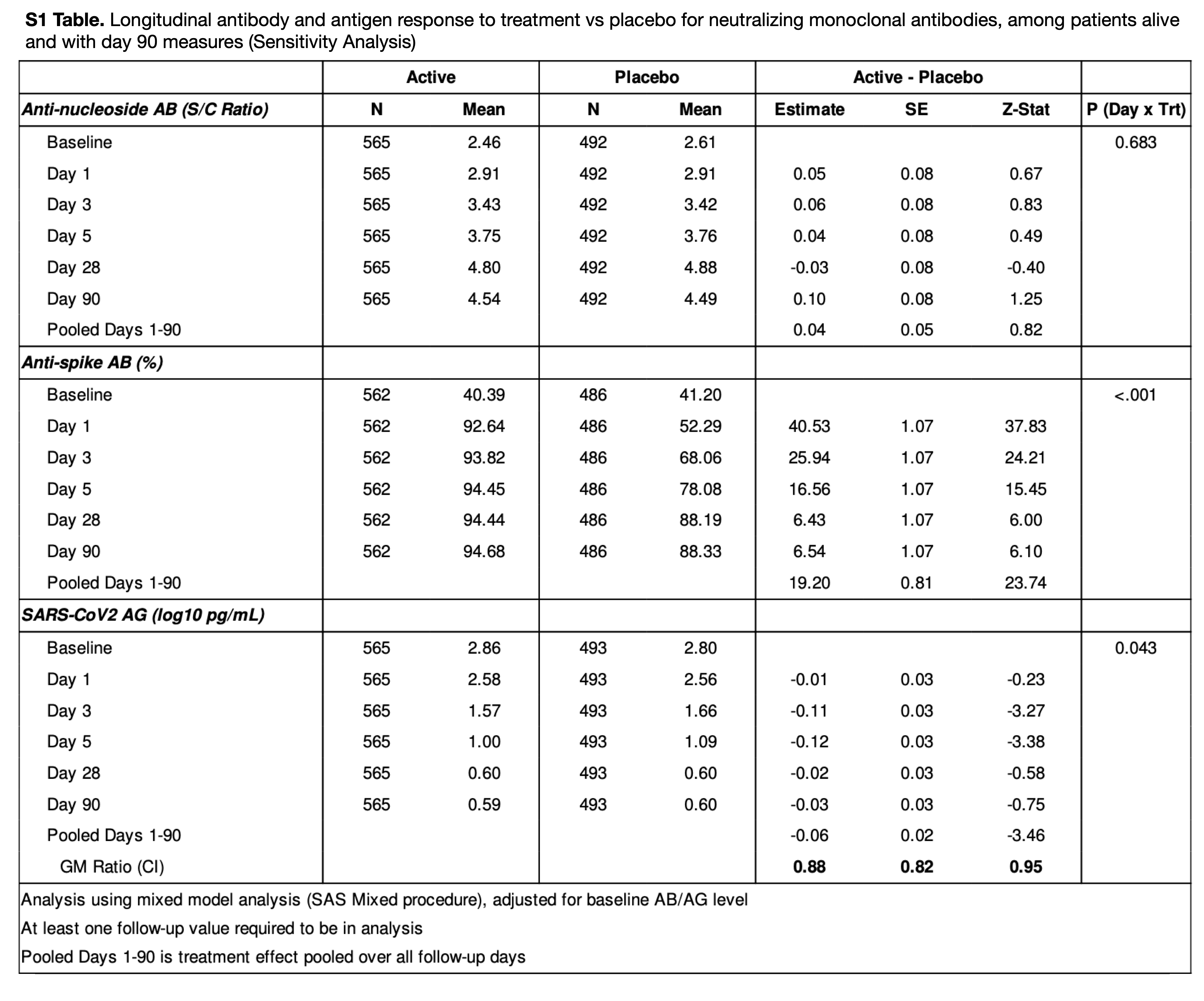

Supplement: S1 Table — Analysis using mixed model analysis (SAS Mixed procedure), adjusted for baseline antibody/ antigen values. At least one follow-up value required to be in analysis. Pooled Days 1–90 represents the treatment effect pooled over all follow-up days. (TIFF) [file pone.0325561.s008.tiff]

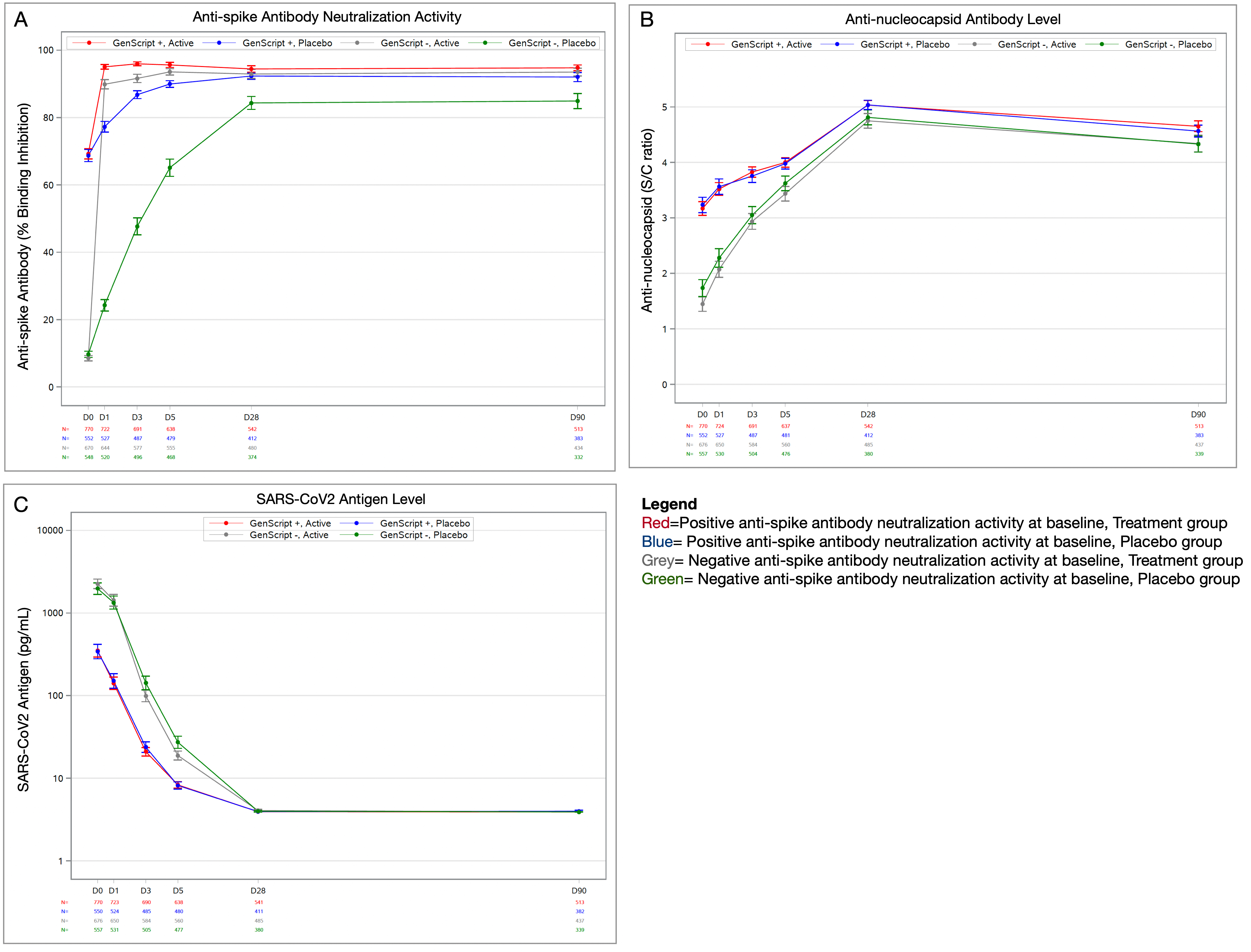

Supplement: S8 Fig — Pooled antibody and antigen responses for randomized controlled trials comparing treatment with all ACTIV-3/TICO treatments (nMAbs, ensovibep, and lufotrelvir) to placebo, by positive baseline anti-spike antibody neutralization activity (>30% binding inhibition). Green and grey lines represent patients with negative anti-spike protein neutralization activity at baseline, while blue and red lines represent patients with positive anti-spike protein neutralization activity at baseline. Panel A: Anti-SARS-CoV-2 spike protein neutralization activity presented as percent binding inhibition (GenScript, Piscataway, New Jersey), Panel B: Total immunoglobulin (all immunoglobulin types) against the SARS-CoV-2 nucleocapsid antigen presented as signal to cut off ratio (BioRad, Hercules, California), Panel C: SARS-CoV-2 nucleocapsid antigen levels presented as pg/mL (Quanterix, Billerica, MA). P-values not displayed. (TIFF) [file pone.0325561.s009.tiff]
