# Supplementary material for: Long-term anti-SARS-CoV-2 antibody trajectories after neutralizing monoclonal antibody treatment
Source: PLoS One. 2025 Jun 18;20(6):e0325561. doi: 10.1371/journal.pone.0325561 (PMC12176123; doi:10.1371/journal.pone.0325561)
Supplement: Attachment — Submitted filename: 20250113- Long-term anti-SARS-CoV-2 antibody trajectories .docx [file pone.0325561.s011.docx]

**Reviewer Feedback and Decision on Manuscript**

Dear Authors

I appreciate the effort invested in conducting this study and recognise its significance in addressing concerns related to the use of neutralising monoclonal antibodies (nMAbs) in hospitalized COVID-19 patients. The study leverages a large dataset from the ACTIV-3/TICO platform and aims to assess the impact of nMAbs on endogenous humoral immune responses. Given the importance of understanding the potential immunological consequences of nMAb treatment, this study has the potential to contribute valuable insights to the field. However, several critical issues need to be addressed to ensure the manuscript accurately represents the findings and effectively answers the research question.

**1. Study Design and Alignment with Study Objective**

1. The study is a secondary analysis of a treatment trial for nMAbs, yet the current framing implies it is a primary investigation into endogenous immune responses.
2. The study design is not adequately designed to address the stated objective of evaluating whether nMAbs impair long-term immune response.
3. The choice of anti-nucleocapsid antibodies as a proxy for endogenous humoral response to SARS-CoV-2 is questionable. Since the nMAbs studied target the spike protein, there is no mechanistic rationale to expect any impact on nucleocapsid-specific responses. Why was this measurement was used to infer immune suppression effects of nMAbs.

**2. Abstract Accuracy and Clarity**

1. The abstract suggests that all randomized participants were tested, which is misleading. In the results section, it is stated that some participants died before follow-up. This needs to be addressed for clarity and accuracy.
2. The term "Day x interaction" in the results section is unclear and does not provide meaningful information. A more precise statistical description should be used.
3. The conclusion in the abstract states that nMAbs did not impair endogenous humoral immune response, which does not align with the study design. Since nMAbs were anti-spike, they were not expected to affect nucleocapsid-specific responses, making the conclusion misleading. This needs to be rephrased to avoid overstating the findings.

**3. Presentation of Study Groups and Data Interpretation**

1. The study groups would be better visualised using a flow diagram, the current description makes it difficult to follow how participants were selected, randomized, and analyzed.
2. Lines 169-170 mention that the different mAbs studied have varying half-lives ranging from 20 to 90 days. Thus, bundling these under a single treatment group introduces heterogeneity, making it difficult to interpret long-term immune response comparisons. Consider analyzing them separately.
3. The placebo group's relevance in the analysis is unclear. If the objective is to assess changes in endogenous immune response due to nMAb treatment, a before-and-after study where each participant serves as their own control might have been more appropriate. The rationale for using a placebo should be explicitly stated.

**4. Statistical and Methodological Concerns**

1. It is unclear whether there was any testing for reinfection during follow-up. If new infections occurred post-treatment, they could confound results related to endogenous immune response.
2. In lines 271 onwards, it is expected that antibody levels would rise in the nMAb group since participants were infused with these antibodies. The novelty of reporting this as a study finding is unclear, the study should focus more on comparing endogenous responses.
3. Lines 284-286 suggest that anti-spike antibodies reduced nucleocapsid antigen levels, but anti-spike antibodies are not expected to have any direct effect on nucleocapsid antigen clearance. This requires further explanation or reconsideration.

**5. Interpretation of Results and Conclusions**

1. The statement that nMAbs "did not impair host response to the target antigen or other viral antigens" (line 318) is not supported by the study design. Since nucleocapsid was not a target of the nMAbs, its response cannot serve as an indicator of whether nMAbs impaired endogenous immunity.
2. The discussion should explicitly acknowledge that hospitalized patients may already be mounting an adaptive immune response by the time they receive nMAbs, making it difficult to assess whether nMAbs suppress early immune responses.
3. The exploratory epidemiologic analysis results need more careful interpretation. The observed differences in long-term anti-nucleocapsid responses by baseline antibody positivity, immunosuppression, and oxygen status may be influenced by confounding factors. These findings should not be overstated.

**6. Structural and Formatting Issues**

1. Lines 254-269 primarily describe the study population and should be moved to the Methods section rather than appearing in the Results section.
2. The limitations section should acknowledge the use of nucleocapsid antibodies as a surrogate for endogenous response to anti-spike antibodies.

The study design does not align well with the research question, and key methodological concerns need to be addressed.
